# The Development of Metaphor Comprehension and Its Relationship with Relational Verbal Reasoning and Executive Function

**DOI:** 10.1371/journal.pone.0150289

**Published:** 2016-03-08

**Authors:** Nuria Carriedo, Antonio Corral, Pedro R. Montoro, Laura Herrero, Patricia Ballestrino, Iraia Sebastián

**Affiliations:** 1 Departamento de Psicología Evolutiva y de la Educación, National Distance Education University (UNED), Madrid, Spain; 2 Departamento de Psicología Básica 1, National Distance Education University (UNED), Madrid, Spain; 3 Facultad de Educación, Camilo José Cela University, Madrid, Spain; The University of Nottingham, UNITED KINGDOM

## Abstract

Our main objective was to analyse the different contributions of relational verbal reasoning (analogical and class inclusion) and executive functioning to metaphor comprehension across development. We postulated that both relational reasoning and executive functioning should predict individual and developmental differences. However, executive functioning would become increasingly involved when metaphor comprehension is highly demanding, either because of the metaphors’ high difficulty (relatively novel metaphors in the absence of a context) or because of the individual’s special processing difficulties, such as low levels of reading experience or low semantic knowledge. Three groups of participants, 11-year-olds, 15-year-olds and young adults, were assessed in different relational verbal reasoning tasks—analogical and class-inclusion—and in executive functioning tasks—updating information in working memory, inhibition, and shifting. The results revealed clear progress in metaphor comprehension between ages 11 and 15 and between ages 15 and 21. However, the importance of executive function in metaphor comprehension was evident by age 15 and was restricted to updating information in working memory and cognitive inhibition. Participants seemed to use two different strategies to interpret metaphors: relational verbal reasoning and executive functioning. This was clearly shown when comparing the performance of the "more efficient" participants in metaphor interpretation with that of the "less efficient” ones. Whereas in the first case none of the executive variables or those associated with relational verbal reasoning were significantly related to metaphor comprehension, in the latter case, both groups of variables had a clear predictor effect.

## Introduction

The study of the cognitive processes involved in metaphor comprehension has been the subject of intense debate in recent years. One of the main debates has focused on the consideration of nominal metaphors as either “class-inclusion assertions” or analogies. From both perspectives, metaphor comprehension is considered to entail a process of integration of the meaning, which requires reasoning verbally from previously acquired concepts or schemas. However, the two perspectives differ in the type of relational verbal reasoning required. Whereas some authors postulated an underlying process of comparison of the metaphor topic and its vehicle based on analogical reasoning [1–5] or between the knowledge domains referred to by the terms contained in the metaphorical relationship [6], other authors (e.g., [7]) defended an underlying process of categorization or class inclusion that would did not imply any comparison, but rather the construction of an *ad hoc* standard category by the metaphor vehicle. However, the metaphor topic would restrict the attributional process, indicating its relevant dimensions[8].

In the context of this last perspective, it has become clear that metaphor comprehension would imply both the activation of concepts that are relevant to its interpretation and the inhibition or active suppression of those properties or concepts that are irrelevant [9–11]From another perspective, Recanati [12] also noted changes in information accessibility in the process of metaphor comprehension: literal interpretation could be more active in early processing stages, whereas non-literal representations could be more accessible in later stages. According to Recanati [12], two factors affected changes in accessibility: the linguistic context and knowledge of the world. This would not imply that the literal interpretation of the metaphor was necessarily suppressed when the non-literal interpretation was accessed, but that the literal interpretation could remain at some level of activation even after the non-literal interpretation has been accessed (for a similar account, see also [13,14], from the fuzzy-trace framework).

All of these works indicated that to properly understand the metaphor, it is necessary to activate relevant information for its interpretation and to suppress (or reduce the accessibility of) the irrelevant information. This activation/inhibition mechanism would be a general mechanism underlying both attributional and comparison processes [10]. The activation/inhibition process would vary according to the metaphor’s degree of familiarity and also depending on the strength of the contextual bias. Under conditions in which metaphors are presented within a context, contextual information helps to differentiate between relevant and irrelevant information. However, when metaphors are presented in a decontextualized manner, their resolution would be analogous to a problem-solving process in which general cognitive resources are involved [13, 15–17] cognitive resources that might be responsible for individual [18] and developmental differences [19]. It has been proposed that analogical reasoning [20], verbal SAT (Scholastic Assessment Test) scores [19], advancement in formal operational development [21], or general intelligence [22] could play a role in these general cognitive processes, as well as processes related to regulation or attentional control [23], such as mental attention [15] or executive functioning.

### The Development of Metaphor Comprehension

In the developmental arena, it has been assumed that metaphor comprehension depends on cognitive development. The studies that have addressed this topic)[15, 21, 24, 25] point to a progressive development with age. However, developmental studies in metaphor comprehension have yielded inconsistent data due to various theoretical, methodological, and linguistic inconsistencies [26]. Thus, it is difficult to establish a sequence of “stages” in metaphor interpretation. Some authors have postulated three main stages, going from exclusively literal interpretations at the age of 3 to the onset of abstract relational verbal reasoning about metaphorical mappings around age 5 [24]. However, other researchers extend the period of development until 9 to 11 years of age, when the ability to paraphrase metaphors emerges [27]. More recent studies have shown a progressive development in novel metaphor comprehension until adulthood [28].

All of these studies concluded that children do not properly understand metaphors until fairly late in development. However, some recent research places metaphor comprehension much earlier in development, suggesting two processing routes for metaphor comprehension [29]: pretence and meaning extension. Those authors who link metaphor comprehension and production to pretence (e.g., Pouscoulous, [30]) maintain that pretence and metaphor require similar cognitive abilities, and thus, children could master metaphors around 2.5 or 3 years of age. On the contrary, other authors underlined that difficulties in metaphor comprehension rely on difficulties in accepting an unconventional label for a familiar entity (e.g., [31]), delaying this competence to around four years of age.

However, the issue under debate is which mechanisms are responsible for the developmental change. From a Piagetian perspective, metaphor comprehension is considered as a reasoning task constrained by logical abilities. In this sense, Paivio [32] noted that metaphor comprehension implied the integration of analogous elements into a new whole through a process of relational reasoning. In this same line, Gentner [33] found that developmental changes in metaphor comprehension could be explained in terms of a ‘relational shift’ that occurs during development (approximately 6–7 years of age): children under age 7 interpret metaphorical comparisons in terms of object similarity (i.e., attributional/perceptual similarity), whereas older children do so in terms of relational similarity. In a similar account, Nall [34] proposed a developmental progression in understanding metaphors: identification of similarities between objects, understanding of relationships between similarities, and integration of similarities in a new concept. On their part, Johnson and Pascual-Leone [15] propose three main levels in metaphor interpretation—identity, analogy, and predicate—which reflect increasing difficulty in mental processing. They also argue that because mental-processing capacity is limited and increases throughout childhood, it could partly explain developmental improvement in metaphor comprehension.

From the information processing approach, it has been shown that semantic knowledge is a reliable predictor of metaphor comprehension (e.g., [35, 36]). Alternatively, other authors have underlined that metaphor interpretation depends on the role of working memory (WM) or attentional resource constraints (e.g., [15, 23]). Therefore, the issue under debate is whether developmental changes in metaphor comprehension are due to an increase in domain-specific knowledge or to changes in more general cognitive abilities, such as attentional capacity or executive functioning.

### Executive Functioning and Metaphor Comprehension

Given that metaphor comprehension demands great abstraction and great attentional effort—and, therefore, requires high levels of control and cognitive regulation—and that such control has been linked to executive functioning, then executive functioning could be a good candidate to explain these observed developmental differences. Thus, our objective was to study the differential contribution of executive functioning across development—specifically, the executive functions of updating information in WM, inhibition, and cognitive flexibility—to metaphor interpretation in relation to analogical and class-inclusion reasoning.

To our knowledge, there are few previous studies relating executive functioning to metaphor interpretation. Some authors have linked the quality of metaphor interpretation to WM capacity [15, 17, 19, 22, 37], whereas others have postulated that cognitive flexibility is required to select the common attributes of the vehicle and the target term and to shift between literal and metaphoric meanings [36], but inhibition control is required to suppress the irrelevant literal interpretation [9–12, 37]

Recently, Prat et al. [17] found interesting results in adults in a Functional Magnetic Resonance Imaging (fMRI) study. They found that neural correlates of metaphor comprehension and analogy resolution mostly overlap, but only when the processing demands of the task increase do the right hemisphere areas (inferior and middle frontal gyri) implicated in metaphor comprehension become increasingly involved. This could reflect a greater need for more general cognitive processes, such as response selection and/or inhibition. That is, as the processing demands of metaphor comprehension increase, areas typically associated with WM processes and areas involved in response selection were increasingly involved. These authors also found that decreased individual reading skill (which is presumably related to high processing demands) was also associated with increased activation both in the right inferior frontal gyrus and in the right frontopolar region, which is interpreted as less-skilled readers’ greater difficulty in selecting the appropriate response, a difficulty that arises from inefficient suppression of incorrect responses. They interpreted these results in terms of the right hemisphere spillover hypothesis, according to which the right hemisphere becomes increasingly engaged when the processing demands of a language task exceed the resources available in the left hemisphere such that some of the residual processing spills over into the right hemisphere [17].

This proposal accords with Johnson and Pascual-Leone’s [15] findings. They observed that metaphor performance is due both to mental-capacity and to knowledge-based factors. However, in adults—unlike the child sample—variables associated with mental attention did not predict metaphor comprehension because adults have already achieved the fifth mental-capacity level, which is the most advanced.

### Rationale of the Study

The main goal of the present study was to analyse the different contributions of relational verbal reasoning (analogical and class inclusion) and executive functioning to metaphor comprehension across development. As previously mentioned, understanding a new metaphor without a context would require not only the involvement of relational reasoning—which could also be implicated in analogical or class-inclusion reasoning—but also the involvement of attentional control processes related to executive functioning, which allows the suppression of inadequate responses, appropriate response selection, the ability to update information in WM, as well as cognitive flexibility. Thus, we proposed that both relational reasoning and executive functioning should predict individual and developmental differences in metaphor interpretation. However, executive functioning will become increasingly involved when metaphor comprehension is highly demanding, either because of the metaphors’ high difficulty (relatively novel metaphors in the absence of a context) or because of the individual’s special processing difficulties, such as low levels of reading experience or low semantic knowledge.

If relational reasoning was the only factor responsible for metaphor interpretation, we would not expect developmental differences, given that most children have already acquired these abilities by approximately 11 years of age. However, executive functioning abilities continue to develop far beyond adolescence. Thus, if executive processing is responsible for metaphor interpretation, we hypothesize changes across development: when executive functioning is not yet established—that is, at the age of 11—metaphor comprehension should rely more on analogical or class-inclusion reasoning. However, when people can benefit from adequate updating of information in WM, suppression of inappropriate information, and effective shifting between literal and metaphorical meanings—that is, at the age of 15 and far beyond—they will use these attentional control resources to achieve a better interpretation.

## Materials and Methods

### Participants

In this study, 119 participants were divided into three age groups: 11 years (*n* = 39, *M* = 11.4, *SD* = 0.41), 15 years (*n* = 41, *M* = 15, *SD* = 0.48), and young adults between the ages of 21 and 25 (*n* = 39, *M* = 23.4, *SD* = 1.32). The children and adolescents between 11 and 15 years came from public and subsidized schools from all over Spain. None of the children had any diagnosed developmental disorders. All of the young adults had at least mid-college studies.

The bioethics committee of Universidad Nacional de Educación a Distancia (UNED) approved this study as a part of a project funded by MINECO (EDU2011-22699) on January 25, 2011. Adult participants were asked to sign a written informed consent form. Parents or guardians signed the written consent form in representation of their children.

### Materials

The participants were evaluated using two relational verbal reasoning tasks—analogical and class-inclusion reasoning—and with the Remote Association Test (RAT), in a task of metaphor comprehension and in various tests commonly used to study executive functions [38]: (a) *shifting* or cognitive flexibility, that is, the ability to change strategies, attention, or tasks when the subject has to perform multiple tasks, operations, or mental processes; (b) *updating* or the ability to supervise, encode, and select information that is relevant to the task at hand, replacing old information that is no longer relevant with new information that is relevant; and (c) *inhibition* or the ability to inhibit dominant or preponderant information and automatic responses when they are not necessary to perform the task. Within inhibition, following the work of Friedman and Miyake [39], we also differentiated between two inhibitory functions: (1) *response-distractor inhibition*—responsible for actively maintaining task goals in the face of interference, usually coming from external stimuli—and (2) *cognitive inhibition*, that is, *suppression of information in WM*, or the ability to inhibit in WM stimuli that are irrelevant to the goals of the task, and *resistance to proactive interference*, understood as the capacity to inhibit items stored in the long-term memory (LTM) that are no longer relevant for the ongoing task.

The analogical reasoning test, class-inclusion test, RAT, and metaphor interpretation test were administered collectively. The order of the reasoning tests was counterbalanced, but the metaphor interpretation test was always presented last. The executive functioning tasks were also counterbalanced and individually administered, controlled by a computer. Randomization and time for stimulus presentation were controlled by E-Prime software, version 2.0 (Psychology Software Tools Inc.: www.pst-net.com/eprime).

#### Analogical, class-inclusion reasoning and metaphor comprehension tests

Taking into account that the existing literature on metaphor interpretation underscores both the influence of analogical and class-inclusion reasoning, we prepared three parallel tests that included similar terms or concepts to control for the possibility that the difference between them was not due to prior knowledge or vocabulary comprehension.

For example, from two trigger words *"lemon"* and "*sweet*" we constructed:

1. An analogy *“…is to sour as honey is to…”* The participants had to complete two terms of the analogy with the words *lemon* and *sweet*.
2. An item of class inclusion. The participants were asked to say the term or general concept that could include both the words *lemon* and *honey*.
3. A metaphorical expression "*your honey tastes like a lemon"*. The participants had to explain the meaning.

Each of the three tests consisted of 12 items designed in accordance with the above criteria. Two of the authors prepared the metaphors especially for this study.

To assess the familiarity, comprehensibility, and aptness of the various metaphors, we conducted a norming study with 43 different participants, aged 18–40 years. They rated 12 randomly ordered metaphors on three scales of familiarity, comprehensibility, and aptness. Following Blasko and Coninne [40], for the familiarity scale, participants were asked to "… *rate each metaphor … according to how familiar the metaphor seems to you*." For the comprehensibility scale, participants were asked to "*… rate each metaphor … according to how comprehensible the metaphor seems to you*." For the aptness scale, participants were asked to "… *rate each metaphor based on how well you think the metaphor expresses its specific non-literal meaning*." These three scales ranged from 1 to 7 on familiarity (1 = *not at all familiar* and 7 = *very highly familiar*), comprehensibility (1 = *not at all comprehensible* and 7 = *very highly comprehensible*), and aptness (1 = *not at all apt* and 7 = *very highly apt*). The results showed that the metaphors used in this study were low on familiarity (*M* = 2.70, *SD* = 0.92), fairly comprehensible (*M* = 5.08, *SD* = 0.69) and apt (*M* = 5.06, *SD* = 0.37). We detected two metaphors whose score on familiarity was slightly above the mean of the scale (4.2 and 4.3). To discard the effect of these different familiarity rates and as per the suggestions of one of the reviewers, we reanalysed the data excluding these two metaphors. As the results essentially did not vary, we decided to maintain them.

Each metaphor item is scored with a minimum of zero points and a maximum of two. The maximum possible score of each test was 24 points. Three independent judges scored the tests. Inter-rater reliability was .90, calculated by using one of the judge’s scores after a previous discussion on any disagreements until consensus was reached. Given that the literature review indicated that inter-rater reliability based upon consensus estimates should be 70% or higher [41] the reliability of these scores could be considered adequate.

The list of items that make up each test is shown in the S1 Appendix. In the metaphor test, examples of a literal interpretation (scored with zero points) and a metaphorical interpretation (scored with two points) are also shown.

**Remote Association Test (RAT):** This was adapted from the original test by Mednick [42]. The test consists of 16 items in which a word that is related to three given words should be produced. For example, from the words *Bass-Complex-Sleep*, the subject should infer *deep*. This test is commonly used to assess creative thinking, which involves relating apparently unrelated concepts, a process that, given its nature, could also be involved in metaphorical interpretation.

#### Executive function tasks

**Updating task:** We used a Spanish adaptation of the updating information in WM task [43] developed by De Beni and Palladino [44] and adapted by Lechuga, Moreno, Pelegrina, Gómez-Ariza, and Bajo [45]. The task had a total of 24 lists (20 experimental lists and four practice lists), each containing 12 words. The words were names of objects, animals, or body parts of different sizes, and abstract common nouns. Each list included words to be recalled (relevant words), words to be discarded (irrelevant words), and filler words. The number of each kind of word in each list varied depending on the experimental condition. Thus, the number of relevant words in each list varied between three (low memory load) and five (high memory load). The number of irrelevant words varied between two (low suppression) and five (high suppression). Finally, the number of abstract filler words varied from two to seven. Table 1 shows the composition of lists as a function of memory load and suppression. Target words (relevant and irrelevant) were familiar concrete nouns, which referred to body parts, objects, or animals that can be classified by size. Filler words were abstract nouns. The final 24 lists were distributed in four experimental conditions of six lists each. One list of each experimental condition was considered as a practice list. Thus, each experimental condition was composed of five lists. The total number of words to be recalled across all conditions was 80 (practice lists were excluded), 25 in the case of each high load condition, and 15 in the case of each low load condition. For 10 of the lists, the participants were asked to remember the three smallest items (low load condition), whereas for the remaining 10 lists, they had to remember the five smallest items (high load condition). Likewise, for 10 of the lists, participants had to suppress the previously presented items that were no longer the smallest items: two items for the 10 lists included in the low suppression condition and five items for the 10 lists included in the high suppression condition (see Table 1). Participants were instructed to listen carefully to the list and when it was finished, they had to recall the three or five smallest animals or objects on the list. At the beginning of each list, a text message was displayed on a computer screen to indicate the concrete number of smallest items to remember (three or five). Then, a beep preceded the first word of the list. At the end of each list of 12 items, a different beep and a large question mark on the screen asked the participants to recall the three or five smallest items of the current list by verbal response. To continue with the next list, participants had to press the space bar. Thus, during the task, participants had to update words according to a semantic criteria (size) that implied substituting and inhibiting previously presented words that were no longer relevant under variable conditions of maintenance (words to be recalled) and inhibition (words to be discarded or inhibited). An example list is as follows: árbol (tree), autobús (bus), piscina (pool), sofá (couch), cesta (basket), tema (matter), acto (act), flor (flower), dedo (finger), lápiz (pencil), oreja (ear), patata (potato). We used the percentage of correctly recalled words as an updating index.

**Table 1:** Composition of the lists as a function of the experimental conditions.

Low load / low inhibition (5 lists)	Low load / high inhibition (5 lists)	High load / low inhibition (5 lists)	High load / high inhibition (5 lists)
3 relevant items	3 relevant items	5 relevant items	5 relevant items
2 relevant items	5 relevant items	2 relevant items	5 relevant items
7 relevant items	4 relevant items	5 relevant items	2 relevant items

#### Inhibition tasks

**Response-Distractor Inhibition:** We used the go/no-go task and flanker tasks.

*The go/no-go task* was adapted from Christ, Steiner, Grange, Abrams, and White [46]. Two experimental conditions were administered: go and no-go. The no-go stimulus was the red t-shirt of the Spanish national football team, and the go stimuli were six t-shirts of other national football teams (i.e., Germany, Argentina, Brazil, France, Netherlands, and Peru), subtending approximately 4.3° horizontally and 5.3° vertically. The screen resolution was 1024 x 768 pixels. In each trial, one of the t-shirts was centrally displayed. Participants were asked to press the space bar as quickly as possible when any stimulus other than the Spanish t-shirt appeared; in that case, participants had to avoid responding. After an interval of 1,000 ms, a new trial was presented. If a participant responded less than 100 ms after the presentation of a target (an anticipatory error), a visual message (“too fast, you cannot see the t-shirt”) was displayed on the screen. In contrast, if a participant failed to respond within 1,500 ms (an inattentive error), a different visual message (“too slow, respond faster”) was presented. If a participant responded on a no-go trial (a false-alarm error), another visual message (“no response needed when you see the Spanish t-shirt”) appeared. Following 49 go trials (neutral phase), six experimental blocks consisting of 40 trials (30 go and 10 no-go trials) were administered. No-go stimuli were randomly presented in 25% of trials. At the end of each block, a break was offered. The participants were seated approximately 60 cm from the computer monitor. The dependent variable was the proportion of errors in no-go trials.

*Flanker task* [47, 48]. The screen resolution was 800 x 600 pixels. The stimuli consisted of five cartoon fish pointing to the right or the left, subtending a visual angle of 8° x 1.2°, horizontally and vertically, respectively. The fish were blue or pink depending on the condition administered. A typical flanker task was administered in the blue condition, and the participants should respond depending on whether the central fish was pointing to the left or right, while trying to ignore flanker fishes at the same time, by pressing the corresponding left or right key on the keyboard (‘Z’ and ‘M’, respectively) with the index fingers of both hands. A reverse flanker task was administered in the pink condition, in which the participants should respond to the flanker fishes’ direction while ignoring the central fish. In three conditions, the target was flanked by three noise stimuli on each side: (a) in congruent trials, the flanker fish were pointing in the same direction as the central one; (b) in incongruent trials, the flankers pointed in the opposite direction from the central fish; and (c) in neutral trials, the fish to be ignored were replaced with geometrical figures without a left-right defined direction. The participants were seated approximately 60 cm from the computer monitor. Each trial started with a cross-shape fixation; 500 ms later, an array of five fish appeared on the screen and remained until the participant responded. A pause of 500 ms separated each trial. The experiment started with a neutral blue block (32 trials; the first eight were warm-up trials not included in the analysis), continued with a practice blue block in which participants had to respond to the central fish (12 trials) and two experimental blue blocks (24 trials each). Then, the fish changed to pink, and participants were instructed to respond to the flanker fish instead of to the central fish. Similar to the blue condition, a neutral pink block (32 trials, the first eight were warm-up trials), a practice pink block (12 trials), and two experimental pink blocks (24 each) were successively administered. Finally, both central (blue) and flanker (pink) conditions were combined in the same blocks, with the fish’s colour cueing the target stimuli in each trial. A practice block (24 trials) and two experimental blocks (48 trials each) were applied under this alternating condition. Auditory and visual feedback were provided in a cartoon-like fashion to sustain a high attentional level. Participants were instructed to respond as quickly as possible while making as few errors as possible. The dependent variable was the mean reaction time (RT) in the incongruent condition in the flanker block.

**Cognitive inhibition:** We used the updating task described above. As indexes of cognitive inhibition, we used the proportion of same-list intrusions and the proportion of previous-list intrusions, which, according to De Beni and Palladino [44], tap two kinds of cognitive inhibition: suppression of information in WM and resistance to proactive interference, respectively.

**Shifting task:** This was measured with the third block of the flanker task, in which central and flanker conditions were combined so that, in some trials, participants had to switch attention from centre to flanker and vice versa. The dependent variable was the mean RT in attentional change trials.

## Results

Following Friedman et al. ([49], RTs < 200 ms were eliminated. For the shifting task, RTs for trials immediately following errors were also excluded. To obtain the best measure of central tendency for each condition, we applied a within-subject trimming procedure that is robust to nonnormality [50].

### Analysis by age

Table 2 shows means and standard deviations of the relevant variables for the different age groups. To test the age effect, several ANOVAs were performed with age as an independent variable and variables related to executive functioning, metaphor interpretation, analogical reasoning, class inclusion, and remote association measures as dependent variables. All *p*s *<* .001, and Bonferroni correction was applied to multiple comparisons, unless otherwise stated. The results showed a significant effect of age for all variables related to executive functioning except for the go no-go task: a) for updating information in WM, *F*(2, 116) = 14.05, η^*2*^ = 0.20; b) for inhibition: for response-distractor inhibition, *F*(2, 116) = 16.14, η^*2*^ = 0.22, and for the two measures of cognitive inhibition—inhibition of information in WM, *F*(2, 116) = 9.65, η^*2*^ = 0.14, and resistance to proactive interference, *F*(2, 116) = 10.33, η^*2*^ = 0.15; and c) for shifting, *F*(2, 116) = 10.58, η^*2*^ = 0.15.

**Table 2:** Means and (standard deviations in parentheses) of the relevant variables^a^.

Variables	11-year-olds	15-year-olds	Young adults
**Updating**			
low load	.81(.15)	.89(.13)	.92(.07)
high load	.76(.15)	.86(.10)	.87(.09)
low suppression	.82(.13)	.89(.78)	.90(.07)
high suppression	.75(.16)	.85(.14)	.87(.09)
overall	.70(.13)	.81(.12)	.84(.09)
**Inhibition**			
**Suppression of information in WM**			
low load	.16(.14)	.08(.11)	.06(.07)
high load	.19(.13)	.11(.09)	.10(.08)
low suppression	.14(.11)	.07(.06)	.07(.05)
high suppression	.21(.15)	.13(.13)	.10(.08)
overall	.16(.12)	.09(.07)	.08(.05)
**Resistance to Proactive interference**			
low load	.04(.04)	.02(.03)	.02(.03)
high load	.04(.04)	.03(.04)	.03(.03)
low suppression	.05(.04)	.03(.04)	.03(.03)
high suppression	.04(.04)	.02(.03)	.03(.03)
overall	.20(.13)	.11(.08)	.11(.08)
**Response-distractor interference**			
Flanker task	679 (174)	560 (171)	475 (128)
Go/no-go task	.81(.11)	.83(.09)	.85(.09)
**Shifting**	1034 (254)	837 (159)	847 (218.)
**Remote assoc.**	9.19(9.71)	13.82(13.64)	28.20(22.91)
**Class inclusion**	56.62(14.74)	60.52(18.21)	59.62(17.99)
**Analogies**	55.77(16.88)	68.39(14.34)	71.15(14.16)
**Metaphor**	38.25(18.51)	53.46(17.77)	67.52(19.19)

^a^ Proportions are presented for all variables except for flanker and shifting, for which TRs in ms are provided.

Multiple comparisons showed the same pattern for all these cases: 11-year-olds performed worse than 15-year-olds and young adults, but no differences were found between 15-year-olds and young adults. The same pattern was also found in analogical reasoning, *F* (2, 116) = 11.46, η^*2*^ = 0.17. In the case of metaphor interpretation, age differences were found among all age groups, *F*(2,116) = 24.45, η^*2*^ = 0.30, and in the case of the RAT, no differences were found between 11-year-olds and 15-year-olds, but differences were found with regard to young adults for both groups. Finally, no differences among age groups were found for class inclusion.

### Correlational analysis

To analyse the relationship between the different variables and metaphor interpretation, Pearson correlations were computed for each age group (see Table 3). The pattern of correlations varied as a function of age. In 11-year-old children, the only variables that were significantly related (*p* < .05) to metaphor interpretation were analogical reasoning (*r* = .44), class inclusion (*r* = .32) and remote associations (*r* = .29), and there was no significant correlation with measures of executive functioning. At age 15, the significant relationship between metaphor comprehension and analogies (*r* = .47) and class inclusion (*r* = .51) remained, but we also observed significant correlations with some measures of executive functioning: updating in all the experimental conditions (*r* = .36), with the highest correlation occurring in the high cognitive demand condition (*r* = .40), suppression of information in WM in high suppression conditions (*r* = -.37), and resistance to proactive interference in conditions of low inhibitory demand (*r* = -.42).

**Table 3:** Correlations among executive functioning and relational verbal reasoning variables with metaphor interpretation for the three age groups.

Variables	11-year-olds	15-year-olds	Young adults
**Updating**			
low load	.05	.31*	.12
high load	.04	.26	.03
low suppression	.10	.14	.02
high suppression	00	.38**	.10
overall	.21	.36*	.37**
**Inhibition**			
**Suppression of information in WM**			
low load	.00	-.34*	-.04
high load	.00	-.23	.07
low suppression	-.06	-.13	.23
high suppression	.04	-.37**	-.09
overall	.06	-.24	.09
**Resistance to proactive interference**			
low load	-.03	-.24	-.23
high load	-.13	-.31*	-.14
low suppression	-.12	-.42*	-.34*
high suppression	-.03	-.03	.02
overall	.08	-.23	-.05
**Response-distractor interference**			
Flanker task	-.19	.14	-.08
Go/no-go task	.15	.15	-.16
**Shifting**	-.15	.00	-.15
**Remote assoc.**	.29*	-.04	.10
**Class inclusion**	.32*	.51**	.23
**Analogies**	.44**	.47**	.37*

*p < .05;

** *p* < .01

In the case of young adults, the significant correlation of metaphor comprehension/ interpretation with class inclusion disappeared, but correlations with analogical reasoning (*r* = .37), updating in all experimental conditions (*r* = .37), and resistance to proactive interference in low suppression conditions (*r* = -.34) were maintained.

### Analysis of the variance communality

Given that we have multiple predictors of metaphor interpretation, one way to decompose *R*^2^ in multiple regression analysis is to conduct a communality analysis to decompose the percentage of variance of the dependent variable that is uniquely associated with each independent variable and the proportion of the explained variance associated with the common effect of the predictors [51]. This procedure allows us to know the exact contribution of each variable in a regression equation. This analysis was performed for each age group.

#### 11-year-olds

The independent variables (IVs) used were analogies, remote associations, and class inclusion because they were the only ones showing significant correlations with metaphor comprehension (see Table 3). A total of 21.6% of the variance of metaphor comprehension was explained (*p* < .04), with analogical reasoning being the variable with more weight at this age, although only marginally significant (β = .336, *p* < .06).

Analysis of the variance communality (see Table 4) showed that analogical reasoning made a unique contribution of 8.10% to metaphor interpretation, representing 37.50% of the total explained variance. The rest of the interactions of analogies with the other IVs had a much lower weight. The unique contribution of both class inclusions and remote associations was approximately 5% of the total of the explained variance.

**Table 4:** Communality analysis, 11-year-olds.

Predictor variables	*R*^2^	Coefficient	Percent
**Unique analogies**	19	8.1	37.50
**Unique class inclusion**	10	1	4.63
**Unique remote associations**	8.5	1.1	5.09
**Common remote associations, class inclusion**	13.5	0.5	2.31
**Common remote associations, analogies**	20.6	2.4	11.11
**Common analogies, class inclusion**	20.5	4	18.52
**Common analogy, class inclusion, remote associations**	21.6	4.5	20.83

#### 15-year-olds

The IVs used were analogies, class inclusion, resistance to proactive interference, and suppression of information in WM because they showed significant correlations with metaphor interpretation (see Table 3). Updating was not included in the regression analysis because, in a preliminary analysis, its contribution to the total explained variance was zero. A total of 52.6% of the variance of metaphor interpretation was explained (*p* < .001). The variable with the highest weight in the regression was resistance to proactive interference (β = -.389, *p* < .002), followed by suppression of information in WM (β = -.311, *p* < .01) and class inclusion (β = -.389, *p* < .002).

Analysis of the variance communality revealed that the unique contribution of resistance to proactive interference explained the highest percentage of variance (27.95%), followed by the unique contribution of suppression of information in WM (17.49%), both processes related to cognitive inhibition. The unique contributions of the two variables related to cognitive inhibition—resistance to proactive interference and suppression of information in WM—were higher than their interaction with analogical or class-inclusion reasoning (see Table 5).

**Table 5:** Communality analysis, 15-year-olds.

Predictor variables	*R*2	Coefficient	Percent
**Unique analogies**	22.4	2.2	4.18
**Unique class inclusion**	25.6	6.9	13.12
**Unique proactive interference**	17.7	14.7	27.95
**Unique suppression**	14	9.2	17.49
**Common proactive interference, analogies**	35.1	1.7	3.23
**Common class inclusion, analogies**	30.9	9.4	17.87
**Common proactive interference, class inclusion**	39.9	0.3	0.57
**Common suppression, analogy**	30.7	1.3	2.47
**Common suppression, class inclusion**	34	1.4	2.66
**Common suppression, proactive interference**	34.1	-2.2	-4.18
**Common suppression, proactive interference, class inclusion**	50.4	-0.1	-0.19
**Common suppression, proactive interference, analogies**	45.7	0.1	0.19
**Common suppression, class inclusion, analogies**	37.9	4.5	8.56
**Common proactive interference, class inclusion, analogies**	43.4	3.4	6.46
**Common suppression, proactive interference, class inclusion, analogies**	52.6	-0.2	-0.38

On the other hand, the unique contributions of the variables related to relational verbal reasoning—class-inclusion and analogical reasoning—were much lower (13.12% and 4.18%, respectively) than their common contribution: the interaction between class inclusion and analogies explained 17.87% of the total variance. Therefore, conjointly, the two relational verbal reasoning variables explained as much variance as suppression of information in WM by itself, but the interaction between the variables of cognitive inhibition and relational verbal reasoning greatly suppressed the effect found.

#### Young adults

The IVs used were analogical reasoning, resistance to proactive interference, and updating because they showed significant correlations with metaphor interpretation (see Table 3). A total of 25.6% of the variance of metaphor interpretation was explained (*p* < .02). The variable with the greatest weight in the regression was analogical reasoning (β = .279, *p* < .07), followed by updating information in WM (β = .225, *p* < .17) and resistance to proactive interference (β = -.208, *p* < .20) (see Table 6). Therefore, none of the independent variables had a significant weight in the regression, and even analogical reasoning fell short of the standard levels of significance.

**Table 6:** Communality analysis, young adults.

Predictor variables	*R*^2^	Coefficient	Percent
**Unique analogies**	13.4	7.3	28.52
**Unique updating**	13.9	4.2	16.41
**Unique proactive interference**	11.4	3.7	14.45
**Common analogies, updating**	21.9	2.7	10.55
**Common proactive interference, analogies**	21.4	0.7	2.73
**Common proactive interference, updating**	18.3	4.3	16.80
**Common analogies, updating, proactive interference**	25.6	2.7	10.55

Analysis of the variance communality revealed that analogical reasoning made a unique contribution of 28.52%; the interaction between resistance to proactive interference and updating made a contribution of 16.80%, whereas the unique contribution of updating was 16.41%.

### Analysis by efficiency

Analysis by age revealed that at age 11, executive functioning did not significantly influence performance of metaphor interpretation. However, it did affect the 15-year-old and young adults groups, especially with regard to the variables related to updating information in WM and cognitive inhibition. Moreover, there were no significant differences in any of the variables of executive functioning between 15-year-olds and young adults, but there were differences in metaphor interpretation. A possible interpretation of this result is that the difference between 15-year-olds and young adults did not reflect the possible individual differences in metaphor processing efficiency. That is, some young adults may have poorer metaphor comprehension than some 15-year-old adolescents. To verify this possibility, a new analysis was performed by dividing the 15-year-olds and young adults not by age, but by good or poor metaphor comprehension. Participants who scored above the median in metaphor interpretation (58.3%) were assigned to the efficient group, and those who scored below the median were assigned to the less efficient group. According to this division, 32 participants (twenty-one 15-year-olds and eleven young adults) were assigned to the "less efficient metaphor processors" group, and 35 (twelve 15-year-olds and twenty-three young adults) to the “efficient metaphor processors” group. To verify that the group division performance actually reflected differential efficiency in metaphor processing, Student’s *t* was performed between the two groups of participants in the variables that made significant contributions to metaphor interpretation. The results showed that the less efficient group of metaphor processors also obtained poorer scores in updating information in WM, analogical reasoning, and class inclusion (all *ps* < .05). However, the difference in cognitive inhibition fell short of the standard levels of significance (*p* < .08).

### Correlational analysis

As Table 7 shows, difficulties in metaphor interpretation were associated with greater effort of executive control related to updating information in WM and cognitive inhibition variables. A high positive and significant correlation was observed between the measures of updating information in WM and metaphor interpretation (*r =* .61), and negative and significant correlations were observed between metaphor interpretation and suppression of information in WM (*r = -*.55) and resistance to proactive interference (*r = -*.41). The correlations with class inclusion (*r =* .53) and analogical reasoning were also significant (*r =* .38). However, there was no significant correlation with any of the variables in the group of efficient processors in metaphor interpretation.

**Table 7:** Correlations among executive functioning and relational verbal reasoning variables with metaphor interpretation for less efficient and more efficient groups.

Variables	Efficient	Less Efficient
**Updating**		
low load	-.04	.60**
high load	-.04	.21
low suppression	-08	.19
high suppression	.00	.51**
overall	.04	.61**
**Inhibition**		
**Suppression of information in WM**		
low load	.14	-.54**
high load	.06	-.30
low suppression	.21	-.12
high suppression	.02	-.55**
overall	.10	-.33
**Resistance to proactive interference**		
low load	-.07	-.40*
high load	-.26	-.37*
low suppression	-.15	-.41*
high suppression	-.22	-.24
overall	.03	-.24
**Response-distractor interference**		
Flanker task	-.29	-.23
Go/no-go task	-.06	.11
**Shifting**	-.11	-.04
**Remote associations**	.18	.15
**Class inclusion**	-.06	.53**
**Analogies**	.04	.38*

*p < .05;

** *p* < .01.

### Communality of variance analysis

Based on the correlational analysis, analysis of variance communality was performed with only the group of less efficient processors. For this purpose, the following variables were selected because they presented higher correlations with metaphor interpretation: inhibition of information in WM under high suppression condition (*r* = -.55), resistance to proactive interference under low suppression condition (*r* = -.41), overall updating (*r* = .61), class inclusion (*r* = .53), and analogical reasoning (*r* = .38) (see Table 7). However, to circumvent interpretational difficulties, we followed the recommendations of Wisler [52]and Mood [53]to group variables when there are many predictors [51]. Thus, we grouped the two inhibition indices—suppression of information in WM and resistance to proactive interference—into a single index that we called *cognitive inhibition*. The correlation of this new variable with metaphor comprehension was *r* = -.60.

The total variance of metaphor interpretation explained by the four resulting variables after the grouping was 47% (*p* < .001). The only variable whose weight was close to significance in the regression was class inclusion (β = .319, *p* < .07). The communality analysis revealed that the highest percentage of variance explained corresponded to the interaction between updating and cognitive inhibition (29.8%), the two variables associated with executive functioning (see Table 8). Class inclusion by itself also explained a considerable percentage (14.68%), but its influence was not summative to the executive functioning variables because the interaction between the three variables was very similar to the unique influence of class inclusion (14.89%).

**Table 8:** Communality analysis. Less efficient metaphor processors.

Predictor variables	R^2^	Coefficient	Percent
**Unique updating**	36.8	2	4.26
**Unique Cognitive inhibition**	35.7	1	2.13
**Unique class inclusion**	28.4	6.9	14.68
**Unique analogies**	14.6	0	0.00
**Common updating, cognitive inhibition**	38.9	14	29.79
**Common updating, class inclusion**	46	1.1	2.34
**Common updating, analogies**	38.7	0	0.00
**Common cognitive inhibition, class inclusion**	45	0.4	0.85
**Common cognitive inhibition, analogies**	37	0	0.00
**Common class inclusion, analogies**	30	1.2	2.55
**Common updating, cognitive inhibition, class inclusion**	47	7	14.89
**Common updating, cognitive inhibition, analogies**	40.1	1.6	3.40
**Common updating, class inclusion, analogies**	46	0.1	0.21
**Common cognitive inhibition, class inclusion, analogies**	45	0.7	1.49
**Common updating, cognitive inhibition, class inclusion, analogies**	47	11	23.40

## Discussion

Metaphor comprehension is a highly complex process, subject to processes of change and development, and requiring high levels of abstraction. The debate in recent years has mainly centred on the consideration of metaphors as analogies or as “class-inclusion assertions.” Although there is evidence in favour of both alternatives, there has also been some evidence relating metaphor interpretation to abilities linked to executive functioning, such as inhibitory processes [9–11], the use of attentionally controlled resources [15] cognitive flexibility [36], and WM [19, 22].

Our main objective was to analyse the contributions of relational verbal reasoning (analogical and class-inclusion reasoning) and executive functioning across development. We postulated that both relational reasoning and executive functioning should predict individual and developmental differences in metaphor interpretation. However, executive functioning will play a supplementary role, especially when metaphor comprehension is highly demanding, either because of the high difficulty of the metaphors (relatively novel metaphors in the absence of a context) or because of the individual’s special processing difficulties, such as low levels of reading experience or low semantic knowledge. Thus, we hypothesized that if relational reasoning was the only factor responsible for metaphor interpretation, developmental differences would not be expected because most children have already acquired these abilities by approximately 11 years of age. However, as executive functioning abilities continue to develop during adolescence and far beyond adolescence, if executive functioning is responsible for metaphor interpretation, we hypothesized changes across development. Thus, when executive functioning is not yet established—that is, at the age of 11—metaphor comprehension should rely more on analogical or class-inclusion reasoning. However, when people can benefit from an adequate updating of information in WM, suppression of inappropriate information, and effective shifting between literal and metaphorical meanings—that is, at the age of 15 and far beyond—they will use these executive resources to better interpret metaphorical sentences.

As expected, our results showed that metaphor interpretation improves across development, as has been shown by other developmental studies [15, 21, 54]. We found that the ability to understand metaphors is present by age 11 and that there is also clear progress from age 11 to age 15, and from age 15 to young adulthood. These results confirmed that metaphor interpretation progresses until adulthood, as reported by Van Herwegen, Dimitriou, and Rundblad [28], especially when the metaphors are unfamiliar and are difficult to understand due to the absence of a context.

Moreover, also as expected, the analysis of the contribution of the different variables to metaphor interpretation varied with age. At age 11, only variables related to relational verbal reasoning were predictive of metaphor interpretation: the variable that accounted for the greatest amount of the explained variance was the unique effect of analogical reasoning (37.5%), but when unique and common effects of analogical reasoning were summed, the explained variance reached 88% of the total variance explained. At the age of 15, relational verbal reasoning measures—analogical and class-inclusion reasoning—were also related to metaphor interpretation. Moreover, different measures of executive functions also made a significant contribution, but only variables related to updating information in WM and to cognitive inhibition—all the measures of updating, inhibition of information in WM, and resistance to proactive interference—so that, conjointly, relational verbal reasoning and executive functioning explained 52.6% of the variance. The analysis of variance communality showed that the variable that accounted for the greatest amount of the explained variance was resistance to proactive interference (27.95%), followed by the unique contribution of suppression of information in WM (17.49%), a percentage of variance similar to that explained by the interaction between analogical and class-inclusion reasoning, which explained 17.87%. Taking into account that the effects are not additive—because the interaction between the variables of cognitive inhibition and relational verbal reasoning considerably suppressed the effect of the unique contribution of resistance to proactive interference—this seemed to indicate that the 15-year-old adolescents use two different alternative strategies to interpret metaphors: either they resolved metaphors using mechanisms of cognitive inhibition, especially resistance to proactive interference, or they did so through analogical and class-inclusion reasoning. We referred to two alternative mechanisms because the interaction of the effects of both inhibitory and reasoning processes suppressed the unique effect attributed to them. Finally, in *young adults*, the studied variables made a lower contribution to metaphor interpretation. Relevant variables—updating information in WM, resistance to proactive interference, and analogical reasoning—explained 25.6% of the variance. The analysis of the variance communality revealed that analogical reasoning made the greatest contribution to the explained variance (28.52%), with executive functioning variables having a much lower weight: the interaction between resistance to proactive interference and updating contributed 16.80%, and the unique contribution of updating was 16.41%. It seems that we again found—as in the case of 15-year-olds—two alternative strategies because the unique effect of analogy decreased when it interacted with the executive functioning variables, whereas the executive functioning variables benefitted from the interaction with other processes.

Therefore, analysis by age could be showing that, given that updating information in WM and cognitive inhibition—both related to executive functioning—are both still developing until late adolescence (and some processes related to inhibition even far beyond that stage) [55, 56], at the age of 11, children could not benefit from WM processes (updating and cognitive inhibition processes) to understand metaphors. Instead, these children rely on relational verbal reasoning to interpret metaphors. However, at the age of 15, when executive functioning is sufficiently consolidated—though still lacking enough reading experience and semantic knowledge—adolescents could benefit from general WM processes. In the case of young adults, contrary to our expectations, although executive processes continued to have a significant effect, their influence decreased, perhaps because young adults use more knowledge-based strategies because of their expected higher reading experience or more developed semantic knowledge. This explanation is consistent with the fMRI results obtained by Prat et al. [17], see also [18], who found that, when indices related to individual differences—such as reading experience—and indices related to WM capacity were used as independent predictors, reading experience (vocabulary size) was more strongly related to neural efficiency in metaphor comprehension in adult participants.

Importantly, our results also corroborated those found by other studies that have not received much attention, such as those of Johnson and Pascual-Leone [15], who also found a different pattern of results in children and in adults in metaphor comprehension. These authors found that unlike the child sample, variables associated with mental attention did not predict metaphor comprehension in adults. In the same line, Prat et al. [17]—using the fMRI technique—found negative correlations between neural activation and WM capacity in executive function and memory brain areas in the easiest experimental condition in adults.

Last but not least, the results of the analysis of the more efficient metaphor processors revealed that not one of the variables considered in this study influenced metaphor comprehension, whereas in the case of less efficient processors, the opposite occurred. There was a significant correlation between metaphor interpretation and updating information in WM, cognitive inhibition (suppression of information in WM and resistance to proactive interference), and relational verbal reasoning measures (class-inclusion and analogical reasoning). All these variables explained 47% of the variance. The analysis of variance communality showed that the interaction between updating and cognitive inhibition explained the greatest proportion (29.8%), and the unique contribution of class inclusion was 14.68%. These results again showed—as in the case of the 15-year-olds and the young adults—the use of two alternative strategies to understand metaphors: either executive control processes or the well-consolidated (since age 11) class-inclusion reasoning were used.

This kind of behavioural dissociation between more and less efficient processors was also observed by Prat et al. [17] at a neural level. These authors found that individuals with higher vocabulary scores and high WM capacity showed less activation in brain areas related to executive functioning. Moreover, when comparing neural bases of analogical reasoning and metaphor comprehension, they corroborated the overlapping of neural and computational components of analogical reasoning and metaphor comprehension. They found that the left lateral prefrontal cortex was activated by relational reasoning and the right lateral prefrontal cortex was also activated when processing demands of metaphor comprehension increased. In fact, when this occurred, an increasing involvement of neural areas related to WM processes and response selection or inhibition, all related to executive functioning, was observed. Likewise, Kazmerski et al. [22]—using the ERPs technique—found that metaphor comprehension was less automatic in participants with lower IQs, a measure that correlated with WM capacity.

Therefore, to process metaphors that require high levels of processing—such as the relatively new metaphors in the absence of context used in this study—demanded from the less efficient processors not only the intervention of relational reasoning but also the supplementary aid of executive functioning, especially cognitive inhibition and updating information in WM. These results also supported those that have previously linked metaphor comprehension to the intervention of inhibitory processes [9–11, 37]. One possibility is that executive functioning could have mediated relational verbal reasoning. However, this did not seem feasible in the light of the results obtained, although it should be corroborated in future research. What we observed in the analysis by age—in 15-year-old adolescents and young adults—and also in the less efficient metaphor processors was the existence of two clearly differentiated strategies, although both were efficient to achieve metaphor interpretation: either processes of relational verbal reasoning or else processes of cognitive inhibition and updating linked to cognitive functioning were used. The analysis of variance communality revealed that they were not complementary, but were alternative strategies, as the use of one of them blocked the effects of the other.

Finally, our results stepped away from the traditional debate over whether analogical or class-inclusion reasoning have more influence on metaphor interpretation. What we have found is that both types of relational verbal reasoning were acceptable strategies to address metaphorical interpretation, but their differential effectiveness depended on the level of development, task difficulty, and, therefore, on the individuals' knowledge of the world to which the metaphor refers.

These results reflect metaphor interpretations of low familiarity items. Thus, it would be desirable to consider different types of metaphors with and without context for future research to more fully address the contribution of executive functioning under different tasks’ demands.

## Supporting Information

S1 Appendix Items used in Metaphors, Verbal Analogies and Class-Inclusion tests. (DOCX)
