# Supplementary material for: The Development of Metaphor Comprehension and Its Relationship with Relational Verbal Reasoning and Executive Function
Source: PLoS One. 2016 Mar 8;11(3):e0150289. doi: 10.1371/journal.pone.0150289 (PMC4783029; doi:10.1371/journal.pone.0150289)
Supplement: S1 Appendix — (DOCX) [file pone.0150289.s001.docx]

**S1 Appendix. Items used in Metaphors, Verbal Analogies and Class-Inclusion tests**

**Metaphors Test**

Below are the 12 metaphors with an example of literal interpretation and a metaphorical example for each one. The examples are taken from the participants' responses.

| **Metaphor** | **Literal interpretation** | **Metaphorical interpretation** |
| --- | --- | --- |
| Your honey tastes like lemon. | You smell like lemons | Someone seems sweet, but is not. |
| The city smells like a town. | The city smells bad | The ways of thinking are old-fashioned. |
| The sob is the laugh of sadness. | It is the way of expressing sadness | When we are glad, laughter emerges, just like when we are sad, a sob is produced (the way of expressing it). |
| I am afraid to put on the wooden suit of the tailor | The splinters hurt | The person is afraid of dying. |
| I hear her/his gaze in my memory. | I remember her/his eyes | The speaker cannot get her/him out of his mind. He is always thinking about her/him. |
| The past is in the basement of my soul. | Whatever happened in the past is over, the important thing is the present | The past is forgotten but it remains. |
| The branch of the tree is a withered flower. | The branch of the tree is always withered | Autumn. |
| The hospital is a prison. | The sick get sad and do not want to stay in the hospital | It is a place that forces you to stay there (hence the similarity with a prison) until you are cured |
| Delinquency is a gangrene. | Nobody does anything about delinquency | Delinquency is a disease of society. |
| It has no hands and it has four feet. | It walks on all fours | It is awkward. |
| Demolished vanity is constructed humility. | It is better to be humble | When we destroy our vanity, we allow humility to surface. |
| In the beginning is the end. | In the before is the afterwards | Everything ends, nothing lasts forever. |

**Verbal Analogies Test**

Below, you will find a series of incomplete verbal analogies, similar to the following:

Engine is to... "car", as heart is to……………….... “body”.

As you can see, the task is to infer the missing terms.

Try to complete the following analogies:

1.......is to EYES as......is to HEARING.

2…...is to SOB as LAUGHTER is to ……………….....

3…..is to AFTER as BASEMENT is to ………………....

4…...is to BRANCH as PETAL is to ………………....

5…..is to SOUR as HONEY is to ………………....

6…..is PRISONER as HOSPITAL is to………………....

7…..is to CONSTRUCT as DEMOLISH is to………………....

8…..is to WOOD as TAILOR is to………………....

9…..is to HAND as LEG is to ………………....

10….URBAN is to..... as .....is to RURAL.

11.... is to BEGINNING, as CLOSING is to ………………....

12-..... is to SOCIETY as GANGRENE is to ………………....

**Class-Inclusion Test**

Below you will find two words that share some common properties. Your task will be to find the general term or concept that includes both words.

For example:

*Airplane- Machinist.....* ***Transportation*.**

In the above example, there is a relationship between the word "airplane" as means of transportation and "machinist" as the driver of another means of transportation other than the previous one, but both words are associated with the more general concept or class that includes them, in this case, the term: **transportation.**

In short, in the same way, try to discover the general term in the following cases, writing the word in the dotted space.

1- EYES - HEARING ........................................

2- SOB - LAUGHTER ........................................

3- PAST - BASEMENT ........................................

4- BRANCH - FLOWER ........................................

5- LEMON - HONEY ........................................

6- PRISON - HOSPITAL ........................................

7- CONSTRUCT - DEMOLISH ........................................

8- WOOD - TAILOR ........................................

9- HAND - FOOT ........................................

10- CITY - VILLAGE ........................................

11- BEGINNING - END ........................................

12- CRIME - GANGRENE ........................................
